# Thyroid Ablation: Minimally Invasive to Added Complexity

**DOI:** 10.1002/wjs.70362

**Published:** 2026-05-16

**Authors:** Kelvin Memeh, Priya H. Dedhia

**Affiliations:** ^1^ Division of Surgical Oncology Department of Surgery Ohio State University Comprehensive Cancer Center and Ohio State University Wexner Medical Center Columbus Ohio USA

Thermal ablation, particularly radiofrequency ablation (RFA), is increasingly used as an alternative to surgery for selected thyroid nodules. While short‐term efficacy and safety are well established, a subset of patients ultimately require thyroidectomy for regrowth, persistent symptoms, or oncologic concern. As utilization expands, surgeons are encountering patients with prior ablation, raising important questions about operative difficulty, complication risk, and diagnostic reliability. However, the available literature remains limited, consisting largely of small series with primarily descriptive findings.

In this context, the study by Zhang et al. provides an important addition to the field, reporting the largest contemporary cohort of patients undergoing thyroidectomy after prior ablation (*n* = 31; 21 thermal ablations, 9 ethanol ablations, and 1 with both), selected under established pre‐ablation diagnostic criteria of two benign biopsies, and incorporating a structured analysis of clinicopathological factors associated with surgical outcomes [1]. While prior studies have established that surgery after ablation is feasible [2, 3, 4, 5], this work begins to define the relationship between ablation‐induced tissue remodeling and perioperative morbidity, although the inclusion of both radiofrequency and ethanol ablation may contribute to variability.

Existing data suggested that prior ablation alters the operative field. Early reports described increased adhesions, distortion of normal tissue planes, and variability in pathological interpretation. In a series of 21 patients undergoing surgery after RFA for papillary thyroid carcinoma, Sun et al. noted a high frequency of perithyroidal adhesion as well as residual disease and nodal metastases, raising concerns about both technical complexity and oncologic adequacy [4]. More recent comparative work has supported these observations. Kuo et al. demonstrated that patients undergoing thyroidectomy after ablation had longer operative times, higher thyroidectomy difficulty scores, and more severe adhesions than non‐ablated controls [3]. Histopathologic studies have also shown that thermal ablation produces a spectrum of changes—including coagulative necrosis, inflammation, and fibrosis—that evolve over time.

The current study builds on this foundation by linking these histologic changes to surgical outcomes. The authors report that fibrosis (sclerosis) is associated with postoperative complications, whereas other parameters, including necrosis, residual viability, nodule size, and ablation‐to‐surgery interval, are not. This represents an extension beyond prior descriptive studies, suggesting that not all post‐ablation changes are equivalent in their impact on surgery. The finding is biologically plausible: fibrosis is the component most likely to obscure dissection planes and tether the thyroid to surrounding structures, thereby increasing operative difficulty and complication risk. However, none of these complications were permanent, indicating that thyroidectomy after ablative therapy, when performed in experienced center, is generally safe even when the surgical planes are compromised.

The clinical implications of this association should be interpreted in context. Although fibrosis cannot be assessed preoperatively, this study evaluated a range of clinical and procedural factors that were not associated with complication risk. While likely limited by sample size, these findings provide a foundation for future studies to identify preoperative predictors of surgical complexity. At present, operative difficulty remains largely an intraoperative determination.

The study also introduces a composite “maturation index” intended to summarize post‐ablation tissue remodeling by capturing the balance between fibrosis, necrosis, and residual viable tissue. This approach represents a thoughtful effort to systematically characterize post‐ablation histologic changes and provides a useful framework for describing tissue evolution, although its clinical applicability will require validation in larger cohorts. This framework also highlights the broader role of histopathologic assessment in understanding how ablation alters the operative field.

Within this context, histopathologic findings—while not the primary focus from a surgical standpoint—offer important insight into the operative challenges observed after ablation. Prior studies show that ablation‐induced changes extend beyond the treated nodule, affecting capsule integrity and adjacent tissue planes and complicating surgical dissection and pathological interpretation. These effects are particularly relevant in follicular‐patterned lesions, where assessment of capsular and vascular invasion is critical. In addition, discordance between pre‐ablation cytology and final pathology, as well as identification of residual disease at surgery, has been reported in multiple series. Together, these findings reinforce that ablation is not definitive therapy in all patients and that delayed surgery may occur in a more complex operative field.

From a clinical perspective, several points emerge. First, thyroidectomy after ablation is feasible but should not be considered equivalent to primary surgery. Even in experienced hands, altered tissue planes and fibrosis may increase operative difficulty. Larger studies in cohorts selected under established pre‐ablation diagnostic criteria (2 benign biopsies) suggest increased surgical difficulty and an increased risk of complications or incidental parathyroidectomy after thyroidectomy following ablation, although the magnitude and types of complications vary across studies [1, 3, 4, 5]. Second, current evidence does not support reliable preoperative risk stratification. The lack of association between commonly available clinical variables and complications underscores the limitations of existing decision tools. Third, these findings support concentration of post‐ablation thyroid surgery in high‐volume endocrine practices, where experience with challenging dissections and routine use of neuromonitoring may help mitigate risk.

Perhaps most importantly, the study highlights the need for careful patient selection when considering ablation. For patients with indeterminate nodules or potential malignancy, the possibility of requiring delayed surgery in a more technically demanding setting should be part of the initial decision‐making process. While ablation offers a minimally invasive alternative for selected patients, its use should be balanced against the potential implications for subsequent surgical management.

Future work should focus on identifying preoperative markers of surgical difficulty. Imaging modalities such as elastography or other techniques capable of assessing tissue stiffness may offer potential surrogates for fibrosis, but require validation. Prospective studies with standardized assessment of operative difficulty and outcomes will also be necessary to better define the magnitude and clinical significance of increased risk.

In summary, Zhang et al. present the largest contemporary series of thyroidectomy after ablation and extend prior work by linking histologic features of tissue remodeling to surgical outcomes. These findings provide important insight into the factors underlying operative difficulty and establish a foundation for future efforts to refine preoperative risk stratification. As thyroid ablation expands, improved patient selection and anticipation of surgical complexity will be essential to minimize delayed surgery and align treatment decisions with long‐term outcomes.

## Author Contributions

**Kelvin Memeh:** writing – original draft, writing – review and editing. **Priya H. Dedhia:** writing – original draft, writing – review and editing, conceptualization.

## Funding

The authors have nothing to report.

## Conflicts of Interest

The authors declare no conflicts of interest.

## Data Availability

Data sharing not applicable to this article as no datasets were generated or analyzed during the current study.

## References

[1] D. Zhang , A. Boero , G. Gazzano , et al., “Fibrosis‐Driven Surgical Risk After Thyroid Nodule Ablation: Quantitative Clinicopathological Determinants of Complications in Post‐Ablative Thyroidectomy – A Retrospective Cohort Study,” World Journal of Surgery (2026), 10.1002/wjs.70300. PMC13070446 41795686

[2] M. Hussein , E. Toraih , P. P. Issa , et al., “From Ablation to Operation: Unraveling the Surgical Outcomes and Complications of Thyroidectomy After Radiofrequency Ablation,” Surgery 175, no. 1 (January 2024): 146–152, 10.1016/j.surg.2023.09.025. 37867100

[3] T. C. Kuo , K. Y. Chen , H. W. Hu , et al., “Surgical and Pathological Challenges in Thyroidectomy After Thermal Ablation of Thyroid Nodules,” Thyroid 34, no. 12 (December 2024): 1503–1512, 10.1089/thy.2024.0281. 39574349

[4] W. Sun , H. Zhang , L. He , et al., “Surgery After Ultrasound‐Guided Radiofrequency Ablation for Papillary Thyroid Carcinoma in 21 Patients: A Retrospective Study From a Single Center in China,” Medical Science Monitor 26 (November 2020), 10.12659/MSM.928391. PMC7690063 33221812

[5] C. Dobrinja , S. Bernardi , B. Fabris , et al., “Surgical and Pathological Changes After Radiofrequency Ablation of Thyroid Nodules,” Internet Journal of Endocrinology 2015 (2015): 1–8, 10.1155/2015/576576. PMC4523654 26265914

